# Possible Risk Factors for Dental Fear and Anxiety in Children Who Suffered Traumatic Dental Injury

**DOI:** 10.3390/dj11080190

**Published:** 2023-08-09

**Authors:** Anka Jurišić Kvesić, Miroslav Hrelja, Željka Lovrić, Luka Šimunović, Bruno Špiljak, Nika Supina, Lara Vranić, Dubravka Negovetić Vranić

**Affiliations:** 1 Private Dental Office, 10000 Zagreb, Croatia; anka.jurisic-kvesic@zg.t-com.hr (A.J.K.); mihrelja@gmail.com (M.H.); zeljka.lovric@gmail.com (Ž.L.); 2 Department of Orthodontics, School of Dental Medicine Zagreb, University of Zagreb, 10000 Zagreb, Croatia; 3 School of Dental Medicine Zagreb, University of Zagreb, 10000 Zagreb, Croatia; nika.supina@gmail.com (N.S.); lara.vranic55@gmail.com (L.V.); 4 Department of Pediatric and Preventive Dentistry, School of Dental Medicine, University of Zagreb, 10000 Zagreb, Croatia; dnegovetic@sfzg.hr

**Keywords:** dental fear and anxiety, parents, children, dental trauma, oral hygiene

## Abstract

Background: Children who undergo painful experiences such as traumatic dental injury (TDI) during their early years are more likely to be at an increased risk of developing dental fear and anxiety (DFA). The purpose of this study was to identify potential risk factors for DFA of these children. Methods: The study participants were 220 parents/caregivers and their children who experienced TDI. Their socio-demographic backgrounds were investigated with the modified WHO Oral Health Questionnaire for Children that included questions about parents’ knowledge and attitudes, while the DFA level was determined using the Children’s Fear Survey Schedule-Dental Subscale (CFSS-DS) and the Simplified Oral Hygiene Index (OHI-S Index) was used to assess oral hygiene status. Results: The confirmed risk factors are parental knowledge, female gender, and degree of oral hygiene and pain in the last three months, while age, type of TDI, presence of soft-tissue injury, and number of subjective complaints were not confirmed. The overall model predicted approximately 54% of variance in DFA, R^2^ = 0.545, F (4.215) = 64.28 *p* < 0.001. Conclusions: These findings emphasise the importance of addressing pain management, improving oral hygiene, and enhancing parental knowledge to mitigate DFA in children with TDIs.

## 1. Introduction

Even though dental care has greatly improved and dental science has advanced, the main issue today is still people skipping dental appointments. Dental fear and anxiety (DFA), which is the main cause of dental avoidance, poses one of the biggest challenges in paediatric dentistry and can ultimately result in deteriorating oral health [1,2,3]. The aetiology of DFA in children is complex and multifactorial and has been attributed to both exogenous and endogenous components [4,5,6,7]. Traumatic dental injuries (TDIs) continue to be one of the most serious oral health issues in children, causing significant pain and distress. They can range from a minor enamel chip to extensive maxillofacial damage involving supporting structures as well as tooth displacement or avulsion [8]. Perhaps no single dental problem has a greater psychological impact on parents and children than the loss or fracture of a child’s front teeth. Primary and permanent front teeth are important not only for aesthetics, but also for phonetics, mastication, supporting tissue integrity, and psychological and mental well-being [9]. If there is pain or a fracture, children may also struggle to enjoy food or maintain proper oral hygiene. However, the psychological, social, and emotional impacts of TDIs vary from person to person, influencing their treatment preferences, coping mechanisms, and eventual recovery. Globally, TDIs during childhood or adolescence are reported to be a common occurrence, affecting up to 30% of young individuals [10,11,12]. Falls at home, school, and during sports activities often lead to TDIs, and children with inadequate lip closure and increased overjet and open bite are particularly prone to such injuries [13,14,15]. The association between child orofacial trauma and physical abuse is worrisome, highlighting the importance of acknowledging the public health implications of TDIs and understanding their impact on the quality of life [16]. Childhood and adolescence are critical developmental periods that present complex challenges in managing TDIs. Dealing with the consequences of childhood TDI involves managing expectations while considering the hidden impacts of the traumatic event. Facial aesthetics, especially for older children and adolescents, play a significant role in self-perception and how others perceive them. Any negative impact on physical appearance due to trauma could cause distress and DFA, further affecting the individual’s quality of life related to oral health, resulting in alterations in oral hygiene habits and heightened levels of DFA among children [17]. Previous research found that children with high DFA have notably higher Decayed, Filled, Missing Teeth (DMFT) index scores compared to those with low DFA levels. Additionally, there is a significant association between a child’s DFA and their dental health, with higher DFA being linked to more severe caries intensity [18]. Consistent monitoring and long-term follow-up, particularly for patients with developing dentition, are essential. Studies [19,20,21] consistently indicate the high likelihood of psychological impacts resulting from traumatic events, leading to an overall negative well-being in affected children and adolescents. In addition, certain TDIs, such as enamel–dentin fractures and periodontal injuries like concussion and subluxation, may be easily overlooked since they do not cause pain to the patient. Consequently, these types of injuries may not significantly raise the child’s level of DFA, unlike TDIs such as avulsion or alveolar bone injury, which can appear visually dramatic to both the parent/caregiver and the child and therefore possibly more easily elevate the DFA level [22]. If parents are informed about the first aid steps to be taken in the event of an accident, they could play an important role in improving the prognosis of TDI in children. It is critical to assess parents’ knowledge level before planning information campaigns. Since the crucial factor in the prognosis of TDI is the period between the injury and the start of treatment, it is important to address the association between parental knowledge, type of TDI, soft-tissue injuries, oral hygiene, and dental fear and anxiety that indirectly affect timely arrival. Therefore, the aim of this cross-sectional study was to identify potential risk factors for the DFA of children with TDIs.

## 2. Materials and Methods

### 2.1. Characteristics of the Study

This cross-sectional study included 220 parents/caregivers and their children who experienced TDI and were referred to the Department of Paediatric Dentistry at the University Dental Clinic in Zagreb, Croatia during June 2020–June 2022. Following referral to the clinic, the parents/caregivers were asked to participate by filling out a questionnaire. Before signing the consent form, all parents/caregivers were fully informed about the study’s purpose. Children with TDI who had no intellectual disability or developmental disorders, no cognitive impairment or psychiatric history, and no serious congenital or acquired oral and maxillofacial deformities met the inclusion criteria.

### 2.2. Scales Used in the Study

The questionnaire that was used in this research was the WHO Oral Health Questionnaire for Children made in 2013 [23] modified in order to collect information on the child’s socio-demographic background (age, gender, and place of residence) among the examined TDI group of patients. The Children’s Fear Survey Schedule-Dental Subscale (CFSS-DS) questionnaire was used to assess DFA. This scale is the most commonly used measuring instrument for determining DFA in children today and was already proved reliable and valid for measuring DFA in Croatian children [24,25,26,27]. Cuthbert and Melamed created it in 1982 [8], and it is based on the Fear Survey Schedule for Children (FSS-FC) instrument for measuring fear presence in younger children [28]. When the CFSS-DS scale is compared to other available psychometric instruments for measuring DFA presence in children, it is shown to have high reliability [25,29,30]. Aside from its widespread use and high reliability, the CFSS-DS scale is easy to use and represents a cost-effective method for DFA evaluation. On the other hand, there are more variations in the analysis of CFSS-DS validity, despite the fact that the results are good (some authors believe that some DFA self-report measures are incapable of distinguishing between general fear and anxiety and DFA) [31]. It assesses dental anxiety by examining various aspects of dental and medical situations. The possible responses to each item ranged from 1 (not afraid) to 5 (very afraid), with total scores ranging from 15 to 75. Children who score 45 on the CFSS-DS test are classified as dentally anxious [32]. The Simplified Oral Hygiene Index (OHI-S Index) was used to assess oral hygiene status using the evaluation criteria described by Green and Vermillion in 1964 [33]. The previous research showed a strong correlation between DMFT and OHI-S index in children 10–15 years old [34]. The OHI-S Index was determined in natural light using a mirror and a probe. Final-year paediatric dentistry residents performed the dental examination, while the calibrated examiner (full professor) repeated measurements (debris and calculus scores) for interrater agreement calculation. The knowledge of parents/caregivers was tested using seven questions. (“Are the front teeth likely to be damaged in dental trauma?” “Will you search for the lost tooth after the bleeding has stopped?” “How urgent do you think it is to seek professional help if a permanent tooth has been knocked out?” “Would you still seek professional advice if the child is not in pain?” “Where are you going to take the child?” (First point of contact)”, “What would you do if you wanted to replant a tooth into its socket but it had fallen to the ground and was covered in dirt?” “What media would you choose to take the tooth?”) [35] while their attitude towards TDIs was observed in 4 questions (“Do you feel guilty about the incident?”, “Were you able to influence the event?”, “Are you afraid that this trauma has a psychological impact on your child?”, “Are you afraid that this trauma has affected your child’s physical appearance?”). The percentage of correct answers divided participants into three groups: inadequate knowledge (up to 2 correct answers, 0–28.57%), partial knowledge (3 to 5 correct answers, 42.86–71.43%), and adequate knowledge (6 or more correct answers, 85.71–100%). Ethical approval for the research was obtained by the Ethical Committee of The School of Dental Medicine, University of Zagreb, Croatia (IRB: 05-PA-30-XVIII-6/2020).

### 2.3. Sample Size

For the sample size calculation in G*Power, F tests-Linear multiple regression: Fixed model, R^2^ deviation from zero was used. In order to ensure an adequate sample, a low effect size (0.1) and high power (0.90) of the test were chosen. Since the model investigated in this study had 8 predictor variables, the total sample size needed to be 199.

### 2.4. Statistical Analysis

The JASP program (version 0.17.2.1., Eric-Jan Wagenmakers, Amsterdam, The Netherlands) was used for statistical analysis. Mean value and standard deviation were used to present descriptive statistics on the average accuracy of answered questions. Given that the variable DFA score failed to confirm the distribution’s normality, the Mann–Whitney and Kruskal–Wallis tests with a post hoc Tukey test were used to compare groups. For graphical representation, box plots were chosen. Linear regression was used to test and estimate the dependence of a quantitative variable DFA score on a set of independent variables such as gender, pain in the last 3 months, parental knowledge, type of TDI, soft-tissue injury, and OHI-S. The results were deemed significant at *p* < 0.05.

## 3. Results

This study involved 220 children who had experienced TDI in some way, 156 of whom were boys and 64 of whom were girls, or 70.91% and 29.09%, respectively. The median age was 10 (8–13) and did not differ between the sexes (*p* = 0.971). Most respondents lived in Zagreb (80%). They most often reported that they brush their teeth two or more times a day (58.18%) and less often once a day (34.55%) or less than that (7.27%). Girls brushed their teeth more often two or more times a day (75%) compared to boys (51.28%) (*p* = 0.003). Additional aids for maintaining adequate oral hygiene were equally used: dental floss (18.18%), interdental brushes (7.27%), and mouthwash (30.91%). As for fluoride in toothpaste, 49.09% used it, 7.27% did not use it, and 43.64% did not even know if the toothpaste they used contained fluoride. The majority of respondents last visited a dentist less than 6 months ago (78.18%), and the reason for the last visit in the majority of respondents was a routine/control examination (58.18%). Children that visited a dentist in the last 6 months showed statistically significant lower DFA compared to ones for whom a longer time had passed (*p* = 0.005). More than two-thirds of parents limited their children’s intake of sugar and sweets, while less than a third read the nutritional values on food packages.

### 3.1. Parents’ Knowledge

The median knowledge of parents was 71.43% (42.86–85.71). Parents residing in Zagreb showed higher knowledge compared to parents outside Zagreb (*p* < 0.001). Also, parents of children who play sports (71.43%, IQR 57.14–86.71) showed statistically significantly higher knowledge than parents whose children did not play sports (57.14%, IQR 28.57–86.71) (*p* < 0.001). The best-answered question was regarding the first place of contact, which was answered correctly in 93.64% of cases, while the worst-answered question was about transport media, answered correctly in only 15.46% of cases (Table 1). Concerning the knowledge shown in the questionnaire, the respondents were divided into the following groups with the indicated shares: adequate knowledge (41.82%), partial knowledge (43.63%), and inadequate knowledge (14.55%). The answers to questions about attitudes, depending on knowledge, are shown in Table 2.

### 3.2. Dental Fear and Anxiety (DFA)

**Figure 1:**
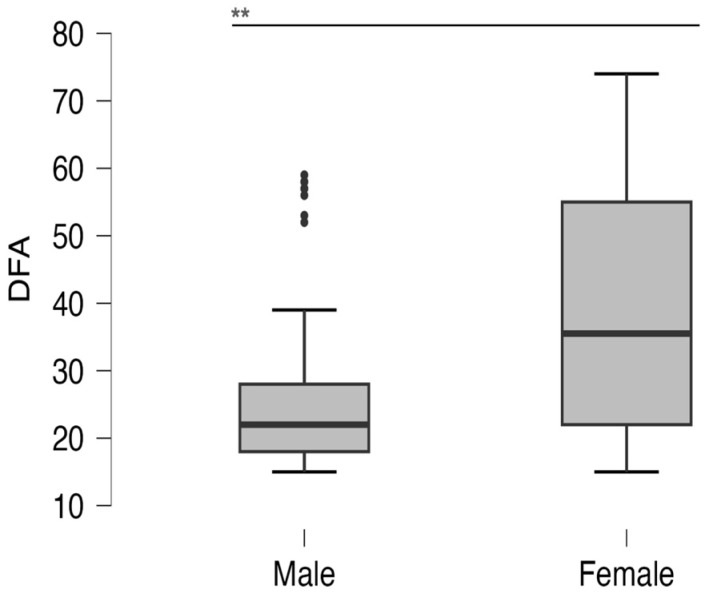
DFA score depending on gender. **: *p* < 0.001.

**Figure 2:**
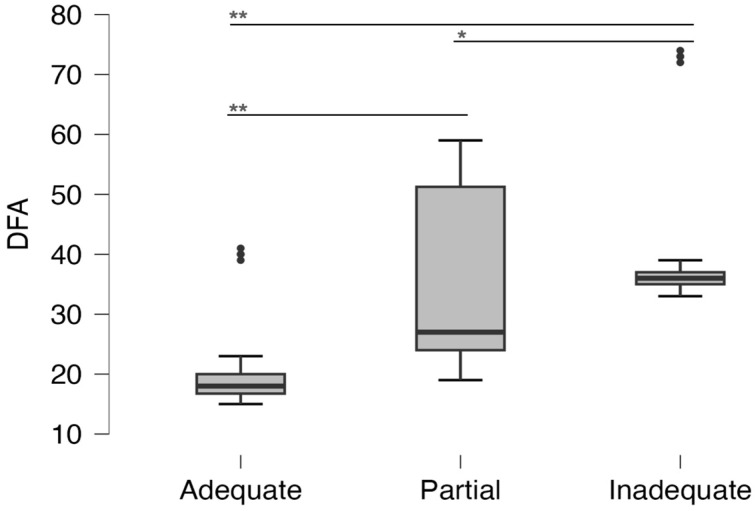
DFA score depending on parental knowledge. *: *p* < 0.005, **: *p* < 0.001.

The median DFA score of all subjects was 23 (19–35) and was statistically significantly higher in girls (Figure 1). There is a weak negative but statistically significant correlation between age and degree of DFA (r = −0.144, *p* = 0.033). Also, a statistically significantly higher DFA score was observed in respondents residing outside Zagreb of 26 (23–33.25), compared to respondents from Zagreb at 22 (18–36) (*p* = 0.042). Children of parents with higher knowledge reported a lower degree of DFA (k = −0.526 *p* < 0.001). The comparison of DFA depending on parents’ knowledge is shown in Figure 2. A weak but statistically significant negative correlation between age and DFA score was also observed (r = −0.144, *p* = 0.033). Also, children who play sports (21.5, IQR 18–28) had a lower DFA score compared to children who do not play sports (30.5, IQR 21–42.25) (*p* < 0.001). Concerning the DFA score, 14.54% of children were highly anxious.

### 3.3. Simplified Oral Hygiene Index (OHI-S)

Interrater reliability was calculated via Cohen’s kappa; the average unweighted kappa was 0.826 (SE = 0.026, 95% CI 0.776 to 0.877), while the weighted kappa was 0.998 (SE = 0.003, 95% CI 0.998 to 0.999). The median OHI-S score was 1.55 (0.775–3.5) and did not differ between genders (*p* = 0.363) or places of residence (*p* = 0.295). There is a moderate positive correlation between OHI-S and DFA (r = 0.310, *p* < 0.001). Considering the OHI-S score, the respondents were divided into groups with the following proportions: good hygiene (43.64%), fair hygiene (26.36%), and poor hygiene (30%). There were no statistically significant differences among the OHI-S scores depending on the level of knowledge of the parents (*p* = 0.080), nor between athletes and non-athletes (*p* = 0.076).

The overall model predicted approximately 54% of variance in DFA, R^2^ = 0.545, F(4.215) = 64.28, *p* < 0.001. There was a significant association between the gender of the child and DFA (B = 11.48, SE = 1.42, *p* < 0.001, 95% CI 8.68 to 14.28), with girls having higher DFA than boys. Additionally, there was an association between experiencing pain in the last 3 months (B = 6.26, SE = 1.55, *p* < 0.001, 95% CI 3.2 to 14.28), OHI-S score (B = 2.53, SE = 0.41, *p* < 0.001, 95% CI 1.71 to 3.34), sum of correct answers of parental knowledge assessment (B = −3.20, SE = 0.32, *p* < 0.001, 95% CI −3.83 to −2.56), and DFA. On the other hand, no significant association was found between number of subjective complaints (B = −0.64, SE = 0.87, *p* = 0.46, 95% CI −2.35 to 1.07), soft-tissue injury (B = −1.14, SE = 1.31, *p* = 0.383, 95% CI −3.72 to 1.44), age (B = −0.081, SE = −0.026, *p* = 0.581, 95% CI −0.368 to 0.207), and DFA. The predicted values of variance in DFA of the first and second step can be found in Table 3.

## 4. Discussion

In this cross-sectional study, the confirmed risk factors are parental knowledge, female gender, degree of oral hygiene, and pain in the last three months, while the unconfirmed factors are age, type of TDI, presence of soft-tissue injury, and number of subjective complaints. Over the past few years, the study designs and target populations have varied considerably, particularly in the scales used for measurement and the age of the children, resulting in a wide range of reported prevalence of DFA in children, ranging from 7.4% to 93.8% [36,37]. According to CFSS-DS, the prevalence of dental anxiety varies greatly in the literature, ranging from 2.4% to 28.3% with variations in different populations and dental situations [36,38,39,40]. In order to select appropriate behaviour management measures, clinicians need to establish a cut-off point for distinguishing children who are at greater risk of dental anxiety. Generally, dental anxiety is measured by categorical cut-off points on a continuous scale. As a result of the balance between specificity and sensitivity in the measuring scale, prevalence estimates vary according to the cut-off values used to define dental anxiety. Several studies have defined children’s dental anxiety cut-off points for CFSS-DS, but the results are not all the same, ranging from 38 to 45. In our study, the median DFA score of all subjects is 23 (19–35), concordant with previous studies in Croatia and the Netherlands [41,42,43] but lower than the findings in Serbia [44]. A recent 2022 survey of the general population showed that the proportion of highly anxious children in Croatia was 12.5% [45]. Although this share was determined using the CDAS scale, we consider this value compatible for comparison considering that in 2017, Haliti and Jurić proved a distinct correlation between the CDAS and CFSS-DS scale [41]. In our study, the proportion of highly anxious children varied from 14.55 to 17.72% regarding the cutoff point (38 or 45), which appears to be a slightly higher proportion than in the general population. However, Negovetić Vranić et al. [46] showed that patients with multiple traumas have a somewhat lower proportion of high anxiety than the general population, potentially due to adaptation to different situations. To our knowledge, this is the first research that attempted to evaluate possible risks such as age, gender, place of residence, child’s sports activity, parents’ knowledge, type of TDI, presence/absence of soft-tissue injury, experiencing pain in the last three months, number of subjective complaints, and oral hygiene status determined via OHI-S in a multifactorial model of dental fear and anxiety (DFA) in children who suffered TDI. The overall model predicted approximately 54% of variance in DFA. Parents’ knowledge was accountable for 20% of variance in DFA in our research. There seems to be a strong connection between these factors, with the result that that the children of parents with higher knowledge have lower DFA. Moreover, parents who live in the city of Zagreb and parents whose children are involved in sport activities also show a higher knowledge and consequently lower DFA. This can be explained by more educational programmes being held in the capital city and sports clubs in a centralised country like the Republic of Croatia. The parents or caregivers of children involved in sports show positive trends in their knowledge about TDIs and their willingness to further enhance their understanding. This can be attributed to increased parental involvement and interest, as their child participates in sports. The lower DFA of children who play sports could not only be explained by the parents’ knowledge, but could also be due to the fact that since they are accustomed to an authoritative figure next to their parents, like their coaches, they may have less anxiety when visiting the dentist. The next factor in this multifactorial model was gender, which is responsible for 19% of the variance. The relationship between dental anxiety and gender is a contentious issue in the literature [47,48]. Girls have significantly higher DFA than boys in our study, which is supported by numerous studies [36,47,48,49,50]. Some studies, on the other hand, found no difference in dental anxiety between girls and boys [38,48,51,52,53,54,55,56,57] and to the best of our knowledge, only one study found higher DFA in boys [58]. These findings can be influenced by factors such as study design, data collection methods, individual willingness to acknowledge emotions, and actual gender differences, emphasising the need to consider gender influences alongside other factors like local culture and family socioeconomic status. The third factor influencing the DFA is oral hygiene status, being responsible for 10.6% of the variance. It has been established that there is a link between anxiety and poor oral health, oral hygiene, and aesthetics [59]. A child’s DFA is predictive of increased dental disease, compromised oral health indicators like untreated dental infection, and higher-risk treatments, leading to negative effects on both the individual and their family’s quality of life, as well as reduced engagement in oral-health-related behaviours [60,61]. Individuals suffering from severe DFA are frequently unable to access standard dental health services [62]. In the study conducted by Uzel et al. [58], no significant correlation was found between children’s oral hygiene habits and their level of dental anxiety. This discrepancy in results could be explained by different indexes for determining oral hygiene, since they utilised the DMFT index and we utilised the OHI-S index. The following factor is pain, which is responsible for 4.6% of the variance. It is well known that children who have toothache or caries are more likely to experience dental anxiety [63,64]. Therefore, pain is a significant factor in the conditioning experiences that lead to the development and maintenance of dental anxiety [6]. DFA is directly linked to the negative sensations resulting from the pain caused by traumatic injuries in children [65]. This negative sensation is a defensive mechanism in an objectively threatening situation, and it may protect the child from further harm [66]. Early-life social and biological factors have long-term consequences for health [67]. A lack of adequate dental treatment leads to more missing teeth and fewer filled and sound surfaces over time [68]. Furthermore, children who undergo painful surgery and are treated under dental general anaesthesia at a young age are more likely to develop dental fear than those who have had positive or neutral dental experiences prior to their first painful surgery [69,70]. In a study conducted by Vanhee et al. [71], it was observed that DFA among paediatric patients exhibited clear bimodal distributions, particularly in response to factors like the sight of a syringe, the sight and taste of blood, and tooth extraction. As a result, high DFA frequently leads to a decrease in oral-health-related quality of life [72]. Mobin et al. [73] revealed that children exhibit heightened fear levels towards “choking” and “injections” compared to other factors such as “the noise of dentist drilling” or “having instruments in their mouth”. This research emphasises the significance of addressing dental fear in children to improve oral health outcomes and overcome the cycle of fear. Although there is a weak negative correlation between age and DFA score, age did not significantly influence the variance in DFA. The reason for this result could be because the most common age group for TDI is 6–12 years, which is not a span large enough to address this association clearly. On the other hand, the literature is in agreement with the DFA decreasing with age [49,51,52,58,74,75,76]. When dealing with stressful situations such as dental treatments, the child’s age is critical [74]. However, few studies have looked at DFA changes over time [52]. DFA increases with age in young schoolchildren, according to one longitudinal study [77]. Furthermore, the age at the first visit has received less attention. Suprabha et al. [78] discovered no link between the age of the first visit (5 years old) and dental fear and anxiety (DFA) and current clinic behaviour. This factor had no effect on the behaviour of school-aged children during dental visits, according to Paryab et al. [79]. The scientific literature has revealed inconsistencies between DFA and treatment experience. Some studies show that patients who do not have DFA have more filled surfaces than those who do, whereas others find no relationship between DFA and fillings in different age groups [80,81,82]. A study found that adolescents with treatment experience had a higher mean DFA score, possibly due to invasive procedures and negative experiences during previous dental visits [83]. A prospective study discovered higher levels of DFA in children with no dental experience and researchers link DFA to fewer dental visits, which can lead to oral health deterioration and the development of additional anxiety disorders [84]. Other studies, however, produce contradictory results, with some claiming that children who visit the dentist more frequently have higher levels of DFA [83]. Brady et al. [85] found an inverse relationship between dental attendance frequency and anxiety in children. Armfield et al. [86] discovered that irregular visits and long intervals are significantly associated with high levels of dental anxiety. According to a study conducted in Iran, 60% of anxious children did not have regular recall dental visits, indicating that irregular dental attendance increases pain and treatment needs and is a risk factor for dental anxiety [79]. Regular dental visits are also emphasised in studies from Europe, Asia, and South America [87,88]. The relationship between the last dental visit and DFA varies greatly [83,86,89]. Children with sporadic attendance and pain frequently require urgent dental care, resulting in high DFA [86]. Longer periods between dental visits increase the risk of oral health deterioration, which may necessitate more invasive dental procedures and higher levels of DFA. Children who had not seen a dentist in the previous year had significantly higher DFA [86], which was also confirmed in our study. Furthermore, in the context of mechanisms for avoiding stressors within dental offices, non-visiting is a global problem of modern dentistry. The formation and development of a vicious circle, associated with previously experienced or assumed stressful situations in the dental office, leads to the onset of DFA, dental behaviour problems, and early avoidance of dental visits. This was confirmed in our study and was also the focus of other studies [41,90,91,92]. This is the core problem of already impaired oral health in childhood, where parents must take on more responsibilities and play more important roles, not only in managing better oral health in their children (proper oral hygiene, anti-cariogenic diet, fluoride usage), but also in cooperating with the dental office to coordinate better cooperative behaviours in their children. As a result, avoidance to visiting the dentist would decrease significantly over time [93,94]. A child who suffers a dental injury must seek treatment from a dentist out of necessity, and thus must accept dental treatment. However, if children see that going to the dentist is not always painful and uncomfortable, they may be able to overcome their dental phobias. The manner in which children are taught to adapt to stressful situations is determined by the individual’s mental characteristics, their level of intellectual development, and the impact of the environment and other people. This supports the idea that cognitive processes and time factors influence fear perception [46]. One of the study’s limitations is that the sample was drawn from a single medical institution. Also, as this study is cross-sectional, it is unable to measure the incidence and investigate the temporal relation between outcomes and risk factors, therefore making it difficult to make a causal inference and interpret identified associations. Expanding the scope of this study to include a greater number of healthcare settings could potentially yield different findings. This is primarily due to the increase in sample size and the inclusion of diverse settings, which may introduce new variables and perspectives. Furthermore, while this article presents numerous contributing factors in the dental environment that have been studied in the literature and provides insight into the possible explanations for the influence of these factors in paediatric patients, there are some factors that have not been examined in the current study, such as previous medical experience, personality traits, genetics, socio-demographics, parental DFA, stress, parents’ satisfaction, dental neglect, and different psychosocial factors that could also affect children’s DFA [18,95,96]. Girls’ higher perception of stress and cognitive styles may increase their risk of developing psychological disorders and anxiety; therefore, salivary biomarkers could be used to measure stress levels, potentially aiding in the diagnosis of DFA [97]. Moreover, understanding parent satisfaction is crucial for providing effective dental care, as satisfied parents are more likely to attend to their child’s dental care, ensuring proper oral hygiene implementation [98]. The use of the CFSS-DS as a measure of DFA, OHI-S index as a measure of oral hygiene status, and the ability to study individual items in the CFSS-DS are all strengths of this research. Furthermore, future studies should adopt a longitudinal and prospective design to gather insights into the long-term effects and potential interventions for reducing DFA. Conducting interventional studies can provide valuable information regarding the most effective methods and strategies to mitigate DFA in children. By addressing these aspects, we can enhance our understanding of DFA and contribute to the development of more targeted and successful interventions in the future. This study’s clinical implications are that patients require psychological preparation for potentially painful or difficult dental procedures. An intriguing possibility are educational programmes for parents/caregivers, since they greatly influence a child’s DFA level.

## 5. Conclusions

This study revealed several factors associated with DFA in children with TDI. These included gender, experiencing pain in the last 3 months, OHI-S score, and parental knowledge.

- Gender: Girls exhibited higher DFA scores compared to boys.
- Parental knowledge: Parents residing in Zagreb and parents whose children play sports demonstrated higher knowledge levels.
- Oral hygiene status: There was a moderate positive correlation between OHI-S scores and DFA, suggesting that oral hygiene practices may influence dental fear and anxiety levels.

These findings emphasise the importance of addressing pain management, improving oral hygiene, and enhancing parental knowledge to mitigate DFA in children with TDIs.

## Figures and Tables

**Table 1:** Descriptive statistics of correctly answered questions. SD: standard deviation.

Question	Mean	SD
Are the damaged front teeth likely to be?	53.6%	5.0%
After control of bleeding, will you search for the lost tooth?	71.8%	45.1%
How urgent do you think it is to seek professional help if a permanent tooth has been knocked out?	88.2%	32.4%
If the child does not have any pain, would you still go for a professional device?	85.7%	78.6%
Where will you take the child? (First place of contact)	93.6%	24.5%
If you decide to replant a tooth back into its socket but it has fallen onto the ground and is covered with dirt, what would you do?	84.5%	36.2%
What media would you choose to take the tooth?	15.5%	36.2%

**Table 2:** Parents’ knowledge depending on attitude.

		Parents’ Knowledge			
Question		Median	Lower Quartile	Upper Quartile	*p* Value
Would you try to replant tooth?	Yes No	42.85% 71.43%	0% 42.85%	89.29% 85.71%	0.449
Do you think you have enough knowledge about TDIs?	Yes No	85.71% 71.43%	57.14% 42.85%	89.29% 85.71%	0.009
Do you think you could affect the accident?	Yes No	85.71% 57.14%	71.43% 42.85%	100% 85.71%	0.003
Did TDI affect the psychological status of your child?	Yes No	85.71% 71.43%	78.57% 42.85%	100% 85.71%	0.002
Did TDI affect the physical appearance of your child?	Yes No	85.71% 57.14%	57.14% 42.85%	85.71% 85.71%	0.005
Are you feeling guilty?	Yes No	85.71% 57.14%	57.14% 42.85%	85.71% 85.71%	0.009

**Table 3:** Linear regression model for DFA.

	Cumulative		Simultaneous	
Variable	R^2^ Change	F − Change	β	*p* Value
Step 1	0.232	32.76 **		
Gender			11.48	<0.001
Pain in the last 3 months			6.26	<0.001
Step 2	0.313	73.82 **		
OHI-S			2.53	<0.001
Sum of correct answers			−3.20	<0.001

** *p* < 0.001.

## Data Availability

Data are available upon request to the corresponding author.
